# Protective Effect of Poria Cocos Polysaccharides on Fecal Peritonitis-Induced Sepsis in Mice Through Inhibition of Oxidative Stress, Inflammation, Apoptosis, and Reduction of Treg Cells

**DOI:** 10.3389/fmicb.2022.887949

**Published:** 2022-05-27

**Authors:** Yu Wu, Dai Li, Han Wang, Xiaojian Wan

**Affiliations:** ^1^Department of Anesthesiology and Intensive Care Medicine, Changhai Hospital, Naval Medical University, Shanghai, China; ^2^Department of Anesthesiology, Bethune International Peace Hospital, Shijiazhuang, China

**Keywords:** Poria cocos polysaccharides, fecal-induced peritonitis, sepsis, oxidative, inflammation, apoptosis, Treg cells

## Abstract

This study was conducted to investigate the potential pharmacological effects of Poria cocos polysaccharides (PCPs) on fecal-induced peritonitis (FIP) mice. Consequently, the fecal peritonitis (FP)-induced septic mice with the higher levels of tumor necrosis factor-α (TNF-α), interleukin 6 (IL-6), IL-1β, malondialdehyde (MDA), myeloperoxidase (MPO), histopathological lesion and bacterial burden, and lower levels of superoxide dismutase (SOD) and glutathione (GSH). Interestingly, PCP pre-treatment reduced inflammatory cytokines and oxidative stress in plasma and spleen and improved the resistance to FIP. Inflammatory infiltration and cell death in thymus or splenic tissue were alleviated with PCP pretreatment. Furthermore, Treg cells were moderated in the spleen with PCP pre-administration. In addition, PCP pretreatment downregulated Annexin-V in the thymus of FP-induced septic mice, and apoptosis of splenic cells was dose-dependent. In conclusion, PCPs have pharmacological and biological effects on FP-induced septic mice, and its molecular mechanism is related to antioxidative, anti-inflammation, anti-apoptosis, and the reduction of Treg activity in splenic cells.

## Introduction

Sepsis is one of the leading causes of mortality and critical illness worldwide (van der Poll et al., 2021) and was considered a major cause of health loss (Cecconi et al., 2018), with nearly 50 million cases globally per year (Rudd et al., 2020). It is reported that 18% of intensive care unit (ICU) patients are suspected of infection (Vincent et al., 2020). At present, sepsis is defined as a life-threatening organ dysfunction caused by a dysregulated host response to infection. Peritonitis is the inflammation of the peritoneum, usually due to a bacterial or fungal infection. Fecal peritonitis (FP) and subsequent sepsis are major problems when admitted to the ICU (Athanazio, 2003). The mortality rate with peritonitis and peritonitis-related sepsis at 28 days was 19.1%, increasing to 31.6% at 6 months (Tridente et al., 2014). Despite the decades of intensive research and more than 200 randomized controlled trials, the underlying mechanisms are still not comprehensively understood and, therefore, there is still no entirely functional treatment that could save lives in patients with sepsis (Ngamsri et al., 2021). Measures remain largely supportive, such as timely antibiotics, source control, resuscitation, and supportive care for organ dysfunction (Evans et al., 2021).

Poria cocos are mainly native to East Asia and Southeast Asia as a kind of medicinal edible fungus, known as “Fu Ling” in Chinese, which has been used in traditional Chinese medicine for more than 2000 years (Wang et al., 2013) and is contained in approximately 15% of traditional Chinese medicine preparations in the Chinese pharmacopeia (Zhu et al., 2018). The bioactive components in Poria cocos include polysaccharides, triterpenoids, fatty acids, sterols, and enzymes (Li et al., 2022). Poria cocos are found in plant polysaccharides, Poria cocos polysaccharides (PCPs), which shows that it accounts for 84% by weight among all constituents in the dried sclerotium (Li et al., 2019), and it is also the main bioactive component in Poria cocos that has extensive biological activities, such as ameliorating phlegm and edema, relieving nephrosis and chronic gastritis, improving uneasiness of minds (Kobira et al., 2012; Wang et al., 2012; Nie et al., 2020), antitumor (Wang et al., 2015; Li et al., 2020), hepatoprotection (Kim et al., 2019; Wu et al., 2019), immunomodulation (Pu et al., 2019), anti-inflammation (Lee et al., 2017), antioxidation (Wang et al., 2016), antibacterial (Wang et al., 2019), and invigorating the splenic functions for immune regulation (Shen et al., 2005). Studies showed that PCP could elicit strong immune activity and a protective effect against immunosuppression (Zhang et al., 2017; Guo et al., 2018), with few side effects and rich resources (Nie et al., 2020).

There are few effective therapeutic measures for sepsis, and refer to the pharmacological properties of PCP, we predicted that PCP can enhance host defenses and may be effective for the prevention of sepsis.

## Materials and Methods

### Materials and Reagents

The PCP was purchased from Beijing Solable Biotechnology (Batch No. SP8930) with a purity of >90%, blending with normal saline to 4 mg/ml paste. Tumor necrosis factor-α (TNF-α) (Catalog: BMS607-3), interleukin 6 (IL-6) (Catalog: KMC0061), IL-10 (Catalog: BMS614), and IL-1β (Catalog: BMS613) ELISA kits were purchased from eBioscience. Glutathione (GSH; Batch No. A006-2-1), superoxide dismutase (SOD; Batch No. A001-3-2), malondialdehyde (MDA; Batch No. A003-1-2), myeloperoxidase (MPO; Batch No. A044-1-1), and other detection kits were purchased from Nanjing Jiancheng Bioengineering Institute. FoxP3 (EPR22102-37, Batch No. ab215206) antibody was purchased from Abcam company, and anti-CD4 FITC (clone: GK1.5, Batch No. 11-0041-82), anti-CD25 PE (clone: PC61.5, Batch No. 12-0251-82), anti-FoxP3 APC (clone: FJK-16s, Batch No. 17-5773-82), Annexin-V (Batch No. 331200), and propidium iodide (PI) (Batch No. BMS500PI) were purchased from eBioscience company.

### Animals and Models

Male C57Bl/6 mice (6–8 weeks, 20 ± 2 g) were purchased from the Animal Experimental Center of Naval Medical University (Shanghai, China) and fed at the animal center of Changhai Hospital Affiliated with Naval Medical University. The ambient temperature was maintained at 25 ± 2°C and relative humidity at 60 ± 10%, with 12 h light/dark cycle. Filtered water and rodent fodder *ad libitum* were freely available to all mice. This study was approved by the Institutional Animal Care and Use Committee at Naval Medical University and abided by NIH animal trial standards.

All the mice were randomly divided into three groups. The PCP-dosed group was taken PCP orally (200 mg/kg, 400 mg/kg) daily for 14 days, according to previous literature (Wu et al., 2018). The PCP-free group and Sham group were intragastrically given with the same volume of normal saline for 14 days. Then, the male C57Bl/6 mice were taken, sacrificed by cervical dislocation, and their cecal feces were fish out, weighed, and diluted. According to our previous experiment and referring to relevant literature (Rosen et al., 2019), mice in the PCP-dosed group and PCP-free group were given the dose of 1 mg/g excrement for 24 h to induce sepsis (fecal-induced peritonitis [FIP]). The mice in the Sham group were given an equal volume of normal saline. Survival rate and Murine Sepsis Score (MSS) were assessed over seven subsequent days.

### Bacterial Burden Determination

Mice with or without PCP pretreatment were euthanized at 24 h post-fecal injection. The blood samples were with dilutions of 10^3^ or 10^5^ in 100 μl saline and coated on a 5% sheep blood agar plate. Similarly, the peritoneum was rinsed with 2 ml of sterile phosphate-buffered saline (PBS) and then the peritoneal lavage fluid was collected and diluted sequentially to 10^5^-fold or 10^7^-fold. Plates were incubated at 37°C for 24 h, and colony counts were expressed as colony forming units per milliliter (CFU/ml).

### Cytokine Analysis

At the end of the experiment, mice were sacrificed by cervical dislocation before blood collection. Blood was taken from the heart and the plasma was prepared by centrifugation at 3,500 rpm at 4°C for 15 min. Circulating cytokine levels (e.g., TNF-α, IL-6, and IL-1β) were measured using commercial ELISA kits (eBioscience) followed by the manufacturer's instructions.

### Morphological Determination

The spleen was fixed with 4% paraformaldehyde for histopathological stains, paraffin-coated splenic sections were stained with hematoxylin and eosin (H&E), and histopathological changes were screened and imaged under an optical microscope. The inflammatory infiltration and cell death of splenic cells were observed in different visual fields in sections. By the immunohistochemical method, the dewaxed splenic sections were sealed with 5% bovine plasma albumin (BSA) at 37°C for 1 h and then incubated with diluted primary FoxP3 (1:1,000). Subsequently, the slices were incubated with manufacture-based Strept Avidin Biotin Complex (SABC) solution and then stained with diaminobenzidine dye and counter-stained with hematoxylin. Finally, the slices are mounted and imaging examined using an optical microscope by experienced pathologists, separately.

### Evaluation of Oxidative Stress

Splenic tissues were homogenized using a homogenizer. Splenic MPO, MDA, glutathione peroxidase (GSH-Px), and SOD activity content and the contents of MPO, MDA, GSH, and SOD for plasma were estimated using the biochemical assay kits (JianCheng Bio Institute, Nanjing, China), according to the manufacturer's instructions.

### Flow Cytometry for the Determination of Treg Expression

The splenic tissue was harvested, and single-cell suspensions were prepared for each sample containing 1^*^10^9^ cells treated with cell lysis buffer (BD Pharm Lyse™, AB_2869057, USA). Then, sections were stained with or without anti-CD4 and anti-CD25 and treated with FIX & PERM medium A and B (Invitrogen, USA). Later, it was also stained with anti-FoxP3. A parallel control group was treated with isotype controls. Flow cytometry was performed using MACSQuant Analyzer (Miltenyi Biotec, Bergisch Gladbach, Germany), and data were analyzed using the Flowjo software version 10.0 (Tree Star, Ashland, OR, USA).

### Determination of Apoptosis in the Thymus and Spleen

Samples of the thymus were harvested, and single-cell suspensions of the thymus were prepared. Annexin-V-positive cells were gated and considered apoptotic events. Cells were incubated with anti-CD3, Annexin V, and PI. A parallel control group was treated with isotype controls according to the manufacturer's recommendations.

The terminal deoxynucleotidyl transferase-mediated dUTP-biotin nick-end labeling (TUNEL) assay was used to assess the degree of splenocyte apoptosis. Formalin-fixed and paraffin-embedded splenic sections were stained using the TUNEL assay. This assay was performed by following the manufacturer's instructions for a commercially available kit (DAB TUNEL Cell Apoptosis Detection Kit, Batch No. G1507-50T, Servicebio, Wuhan, China) and evaluated by immunofluorescence staining. The reaction was stopped after incubation for 1 h at 37°C. The slices were washed followed by the addition of an anti-digoxigenin-peroxidase solution. The sections were then colorized with DAB/H_2_O_2_ and counter-stained with bisbenzamide. Five random fields of view were assessed per section. The apoptotic index for each mouse was calculated as the percentage of TUNEL-positive cells (TUNEL-positive cell number/total splenic cell number × 100%).

### Statistical Analysis

All data were analyzed using the GraphPad Prism version 8.0 software, and normal distribution was used in the Kolmogorov–Smirnov test. Analysis of variance (ANOVA) was used to compare the differences between groups. The Bonferroni test was used as a *post-hoc* test for the correction of multiple comparisons. The final data were expressed as mean ± standard deviation (SD). The log-rank test was performed to determine the significance of difference between the groups in the survival study. Differences between groups were considered statistically significant when *P* < 0.05.

## Results

### Biological Benefits of PCP on FIP-Septic Mice

**Figure 1:**
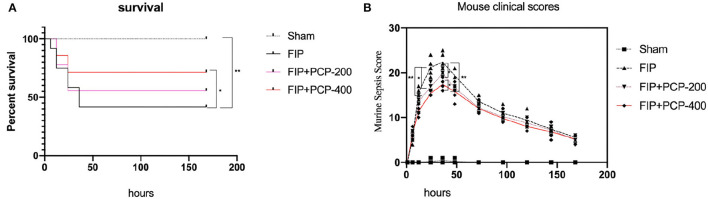
Poria cocos polysaccharide (PCP) pretreatment improved the 7-day survival and Murine Sepsis Score (MSS) of septic mice. **(A)** The survival of mice in the normal group was in good condition and the survival rate of mice decreased linearly after fecal injection. While the mice received PCP, the survival rate increased significantly, but the survival rate had no significant difference between receiving PCP of 200 and 400 mg/kg/day. **(B)** The MSS of experiment mice in each group. The MSS of mice increased significantly at 6 h after fecal injection, the upraise lasts for 24 h, and a peaked at 36 h after treatment. The MSS of PCP-treated mice increased less. Meanwhile, there was no difference in the MSS between PCP 200 and PCP 400 mg-treated mice. ^*^P < 0.05; ^**^P < 0.01, FIP + PCP-200 = FIP with PCP 200 mg/kg/day; FIP + PCP-400 = FIP with PCP 400 mg/kg/day. *n* = 12.

After successfully causing FP, all of the mice moved freely and none needed analgesia for pain immediately. We observed the survival rate and evaluated the clinical scores of mice by incorporating the MSS at 0, 1, 6, 12, 24, 48, 72, 96, 120, 144, and 168 h. The scoring criteria are as follows (Shrum et al., 2014): spontaneous activity, response to touch and auditory stimuli, posture, respiration rate and quality (labored breathing or gasping), and appearance (i.e., degree of piloerection), each of these scores variables between 0 and 4, and the symptoms the severer, the score the higher. As shown in Mouse Scoring Systems after FP for 24 h later, FP-induced septic mice resulted in reducing Mouse Scoring Systems when compared to those in FIP-free mice (*P* < 0.05). As can be seen from Figure 1, the MSS was correlated with the survival rate. A high MSS indicates that mice have severe infection symptoms and are prone to death.

### PCP Reduced Bacterial Burden

**Figure 2:**
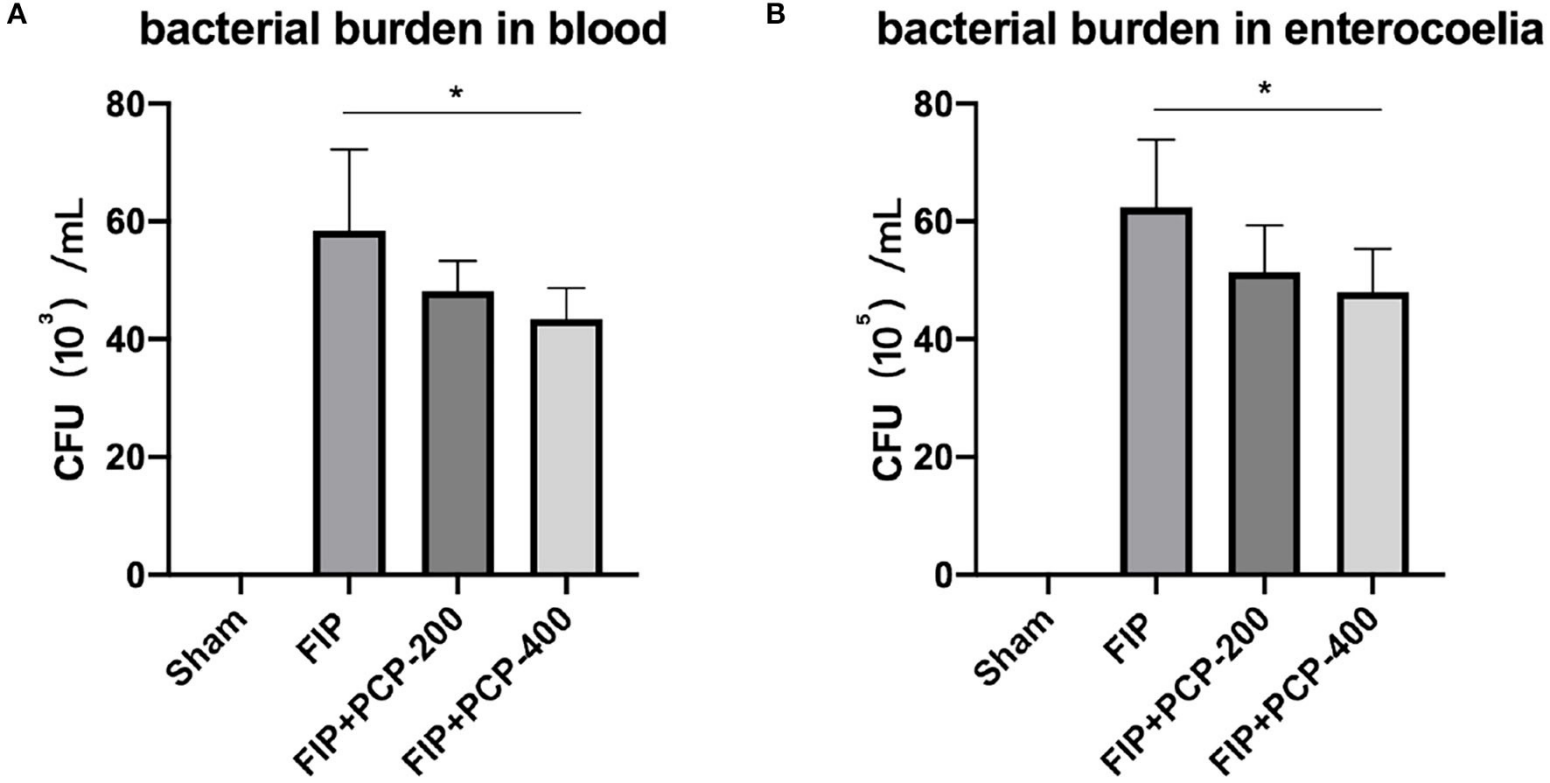
PCP-400 mg/kg/day pretreatment reduced the bacterial burden in peripheral blood **(A)** and at the enterocoelia **(B)**. No significant difference between PCP-400 mg/kg/day and PCP-200 mg/kg/day. All presented data are a composite of three independent experiments. *P < 0.05, *n* = 5 in each group.

The capacity of bacterial clearance was assessed by measuring bacterial burden. The FP-induced septic mice with PCP 400 mg/kg/day pretreatment, bacterial burden levels in peripheral blood, and enterocoelia were reduced at 24 h post-fecal injection (Figure 2).

### PCP Reduced Inflammatory Cytokines in FP-Induced Septic Mice

**Figure 3:**
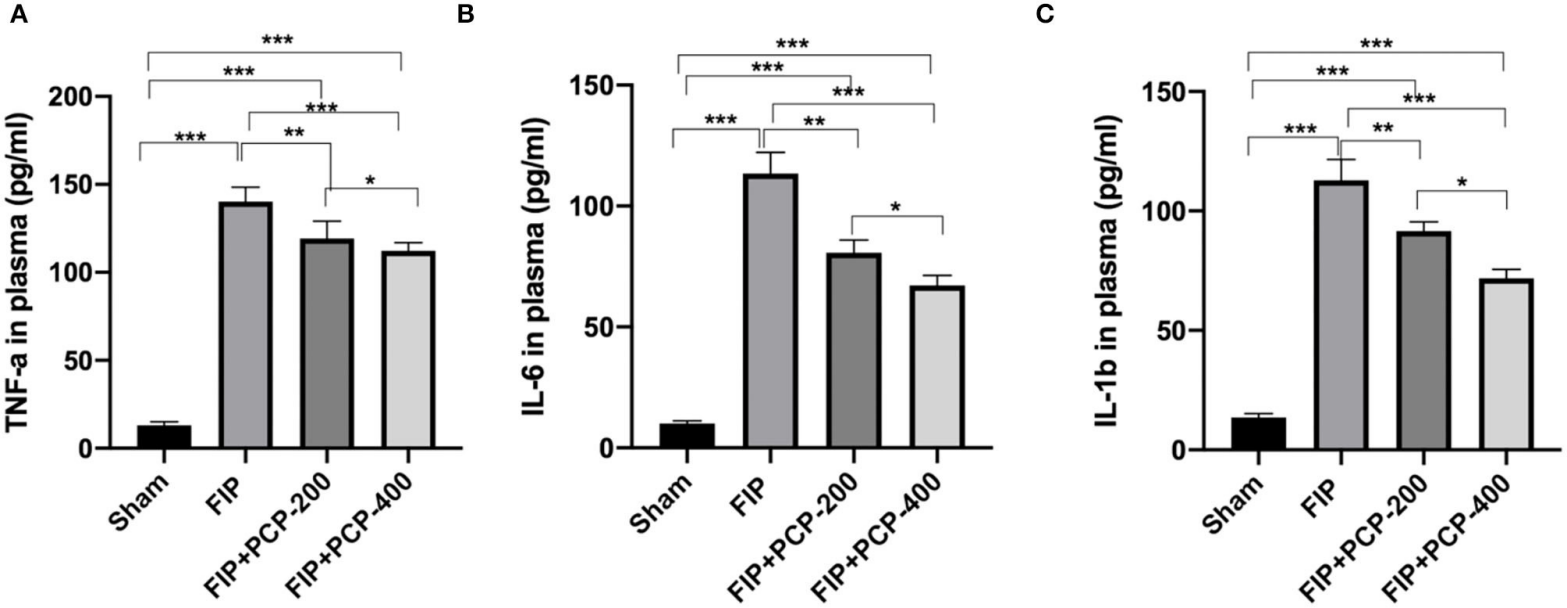
PCP pretreatment inhibited the inflammatory response in FP-induced septic mice. **(A)** TNF-α in plasma in each group. **(B)** IL-6 in plasma in each group. **(C)** IL-1β in plasma in each group. *P < 0.05; **P < 0.01, ***P < 0.001, FIP + PCP-200 = FIP with PCP 200 mg/kg/day; FIP + PCP-400 = FIP with PCP 400 mg/kg/day. All presented data are a composite of three independent experiments. *n* = 5 in each group.

Notably, the FP-induced septic mice had significantly higher levels of TNF-α, IL-6, and IL-1β in plasma compared to the Sham-treated mice (*P* < 0.05). Following PCP pretreatment, these cytokine expressions were suppressed in a dose-dependent manner. The concentration was significantly reduced compared with the FIP septicemia control group (*P* < 0.05) (Figure 3).

### PCP Influenced Oxidative Stress in Fecal Peritonitis-Induced Septic Mice

**Figure 4:**
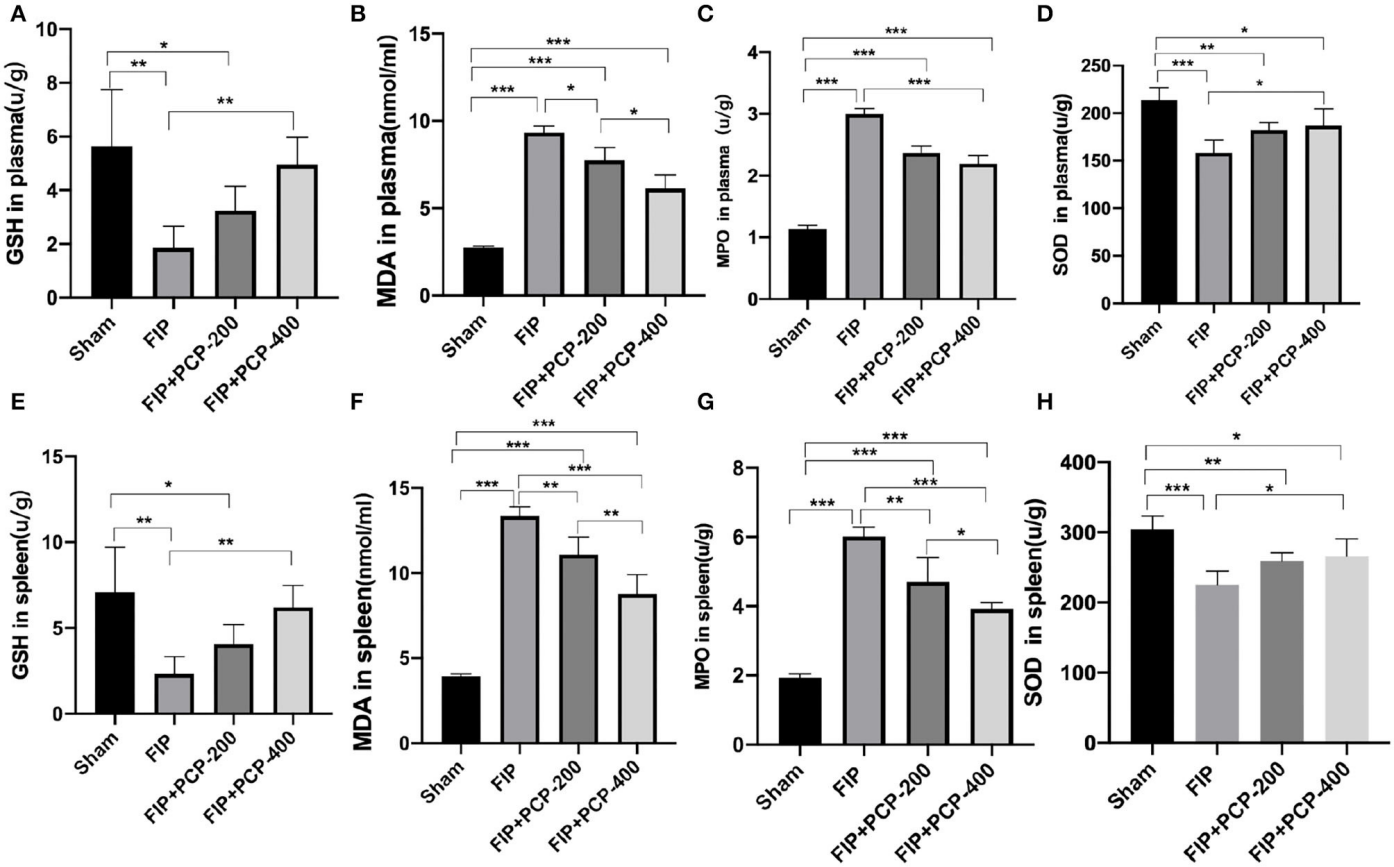
PCP pretreatment inhibited the oxidative stress in fecal peritonitis-induced septic mice. **(A)** GSH in plasma in each group. **(B)** MDA in plasma in each group. **(C)** MPO in plasma in each group. **(D)** SOD in plasma in each group. **(E)** GSH in the spleen in each group. **(F)** MDA in the spleen in each group. **(G)** MPO in the spleen in each group. **(H)** SOD in the spleen in each group. *P < 0.05; **P < 0.01, ***P < 0.001, FIP + PCP-200 = FIP with PCP 200 mg/kg/day; FIP + PCP-400 = FIP with PCP 400 mg/kg/day. All presented data are a composite of three independent experiments. *n* = 5 in each group.

The oxidative stress in plasma and spleen was evaluated by GSH, MDA, MPO, and SOD levels through Elisa kits. The expressions of MPO and MDA levels were higher, while SOD and GSH levels were lower when intervened with feces injection. In contrast, PCP pretreatment reversed these manifestations, as shown in Figure 4.

### PCP Suppresses Histopathological Changes in Fecal Peritonitis-Induced Septic Mice

**Figure 5:**
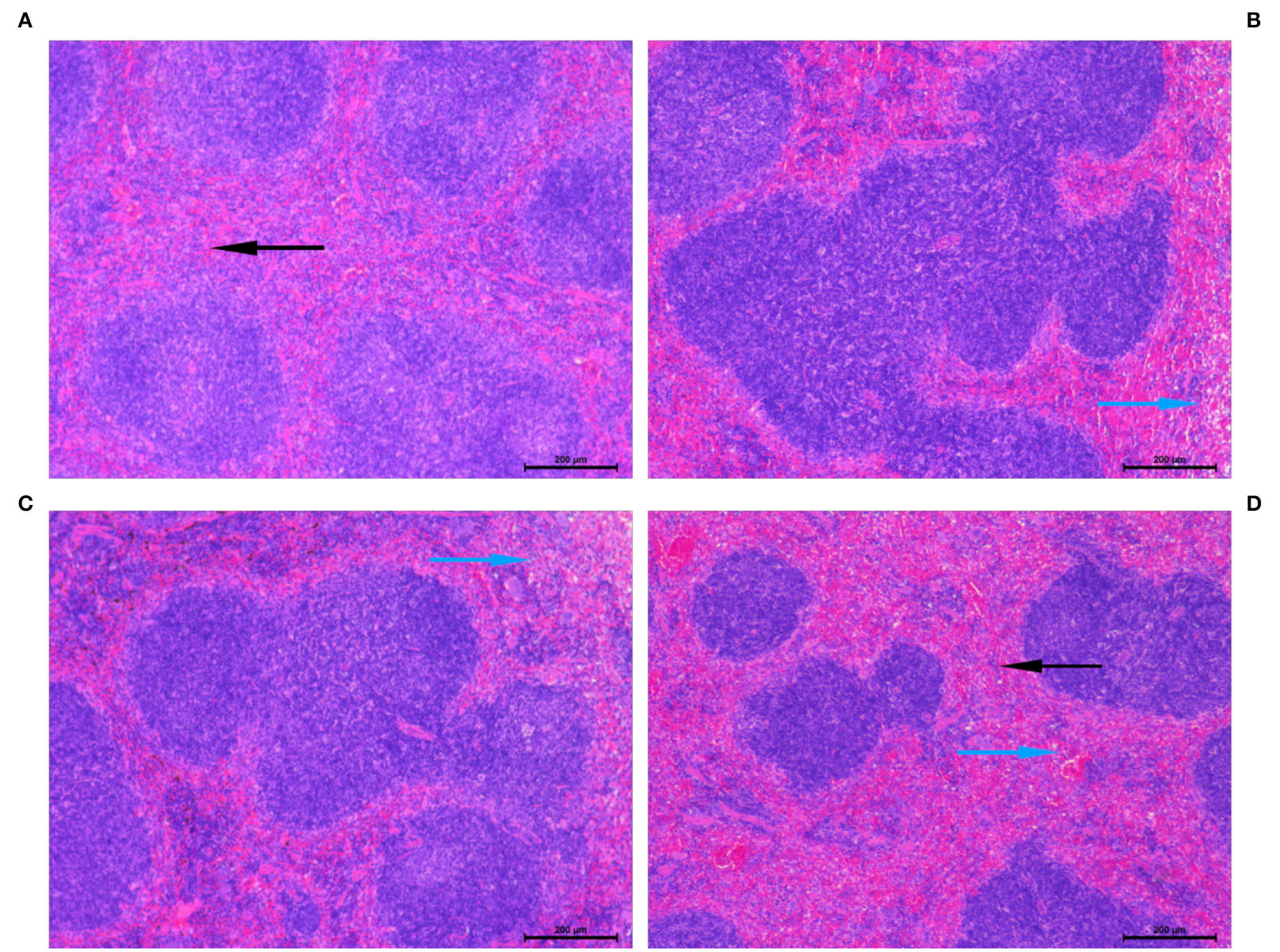
Histopathological lesions were determined in the spleen following fecal peritonitis-induced septic. Representative images showed an improvement in the vital organ injury in mice pretreated with PCP-400. Sections were examined under the microscope at a magnification of 200 × by two separate pathologists. The blue arrows indicate histopathological lesions and the black arrows refer to normal pathological appearance. **(A)** Sham. **(B)** FIP. **(C)** FIP + PCP-200. **(D)** FIP + PCP-400. FIP + PCP-200 = FIP with PCP 200 mg/kg/day; FIP + PCP-400 = FIP with PCP 400 mg/kg/day. All presented data are a composite of three independent experiments. *n* = 5 in each group.

The splenic injury was determined at 24 h post-fecal injection or Sham treatment. As shown in Figure 5, FP induced substantial pathological lesions in the spleen. Pathological changes in the spleen showed vascular leakage, deranged tissue, cytoskeletal damage, necrosis, and inflammatory infiltration and cellular apoptosis following feces infection. PCP pretreatment largely alleviated the pathological lesions as compared to that in the Sham group (Figure 5).

### PCP Reduced Treg Cells in Fecal Peritonitis-Induced Septic Mice

**Figure 6:**
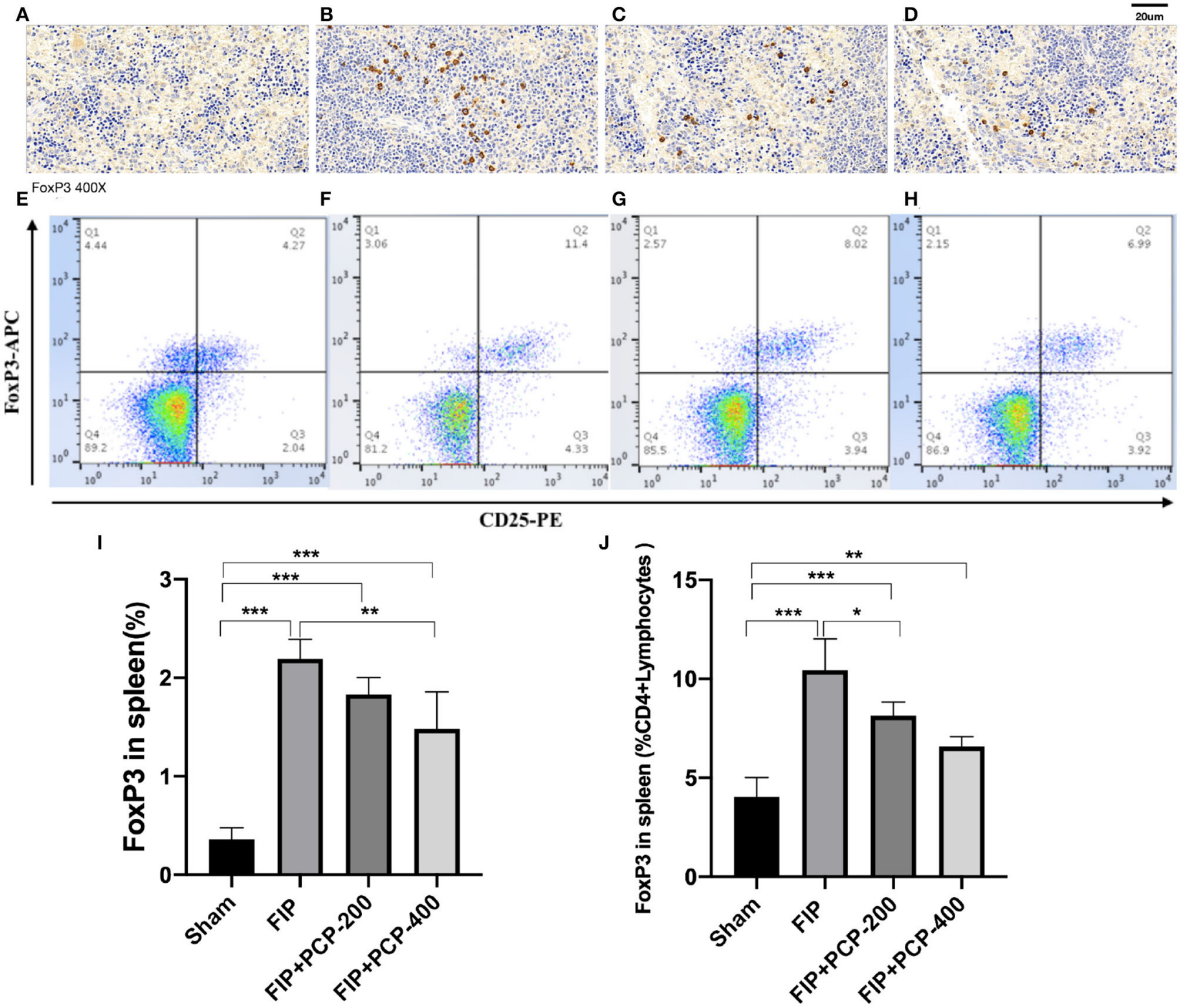
The impact of PCP on Treg cells in the fecal peritonitis-induced septic spleen. PCP pretreatment contributed to decreased FoxP3 labeled cells and CD25^+^FoxP3^+^ cells. **(A–D)** Immunohistochemical analysis. **(E–H)** Flow cytometry analysis. **(A,E)** Sham. **(B,F)** FIP. **(C,G)** FIP + PCP-200. **(D,H)** FIP + PCP-400. **(I)** Treg cells in the spleen. **(J)** FoxP3 positive cells in the spleen CD4+ cells. *P < 0.05; **P < 0.01, ***P < 0.001, FIP + PCP-200 = FIP with PCP 200 mg/kg PCP; FIP + PCP-400 = FIP with PCP 400 mg/kg. All presented data are a composite of three independent experiments. *n* = 5 in each group.

To evaluate the changes in immune function in FIP sepsis mice, we observed the subtypes of splenic cells that fed and non-fed PCP by flow cytometry and immunohistochemical analysis. The results showed that FP increased the Treg cells, and pretreatment with PCP could largely alleviate the expression level of Treg cells as compared to that in the Sham group (Figure 6).

### PCP Regulated Annexin-V and PI in FIP-Septic Mice' Thymus

**Figure 7:**
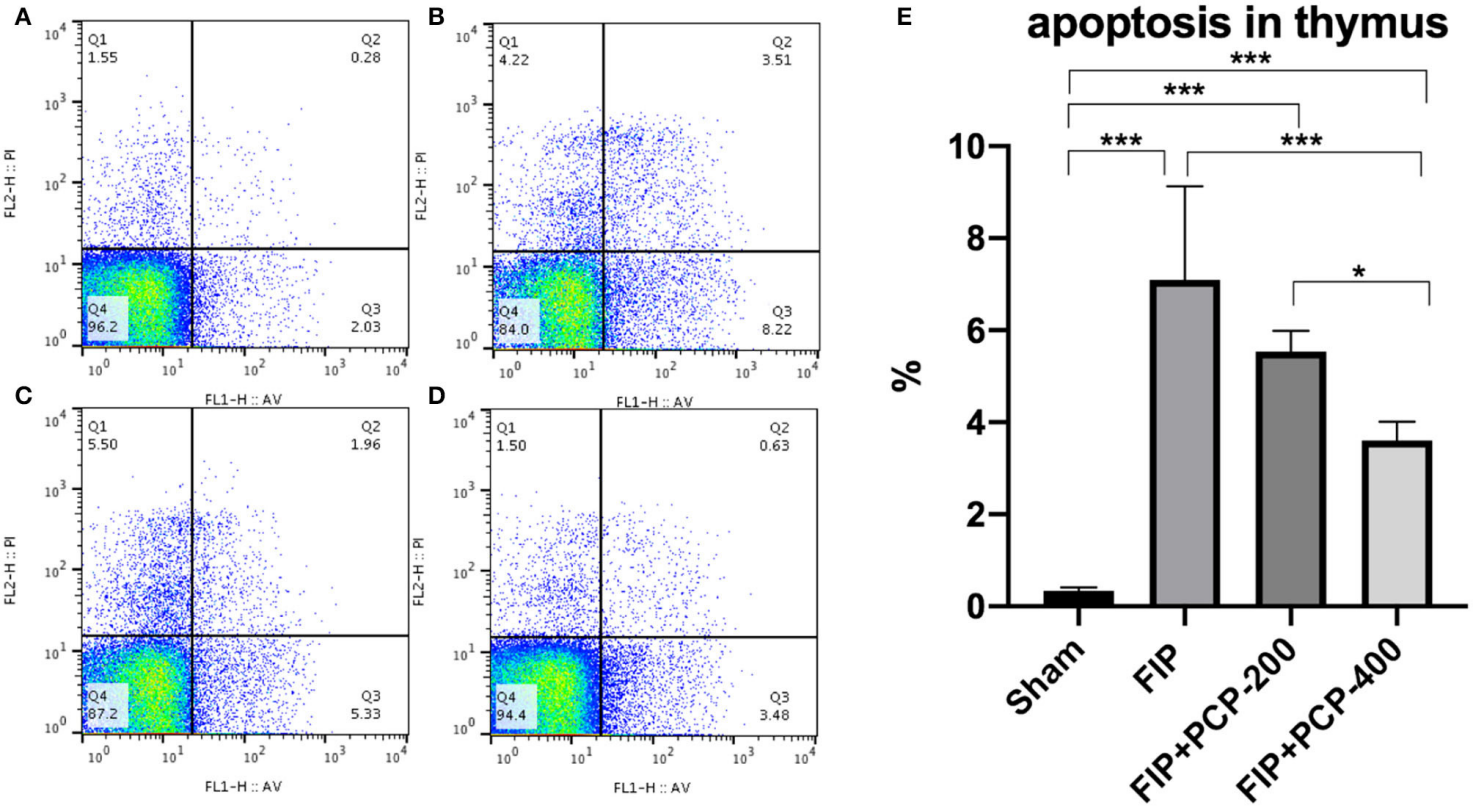
The impact of PCP on apoptosis in the fecal peritonitis-induced septic thymus. PCP pretreatment contributed to decreased Annexin-V, PI. **(A)** Sham group; **(B)** FIP; **(C)** FIP + PCP-200; **(D)** FIP + PCP-400. **(E)** Histogram of four groups. As shown, the differences between FIP and FIP + PCP-400 had a significant difference. *P < 0.05; ***P < 0.001, FIP + PCP-200 = FIP with PCP 200 mg/kg/day; FIP + PCP-400 = FIP with PCP 400 mg/kg/day. All presented data are a composite of three independent experiments. *n* = 5 in each group.

Apoptosis was determined in the thymus at 24 h post-FIP or Sham treatment. Flow cytometry and data analysis were performed to further determine the anti-apoptotic activity of PCP against FP-induced sepsis. The number of Annexin-V-positive T cells was extremely low in the thymus from the Sham group, and sepsis resulted in a marked increase in the number of apoptotic events. Importantly, with pretreatment with PCP, the number of Annexin-V-positive cells in the thymus was decreased in a dose-dependent manner (*P* < 0.05) (Figure 7).

### PCP Relieved Splenocyte Apoptosis in FIP-Septic Mice

**Figure 8:**
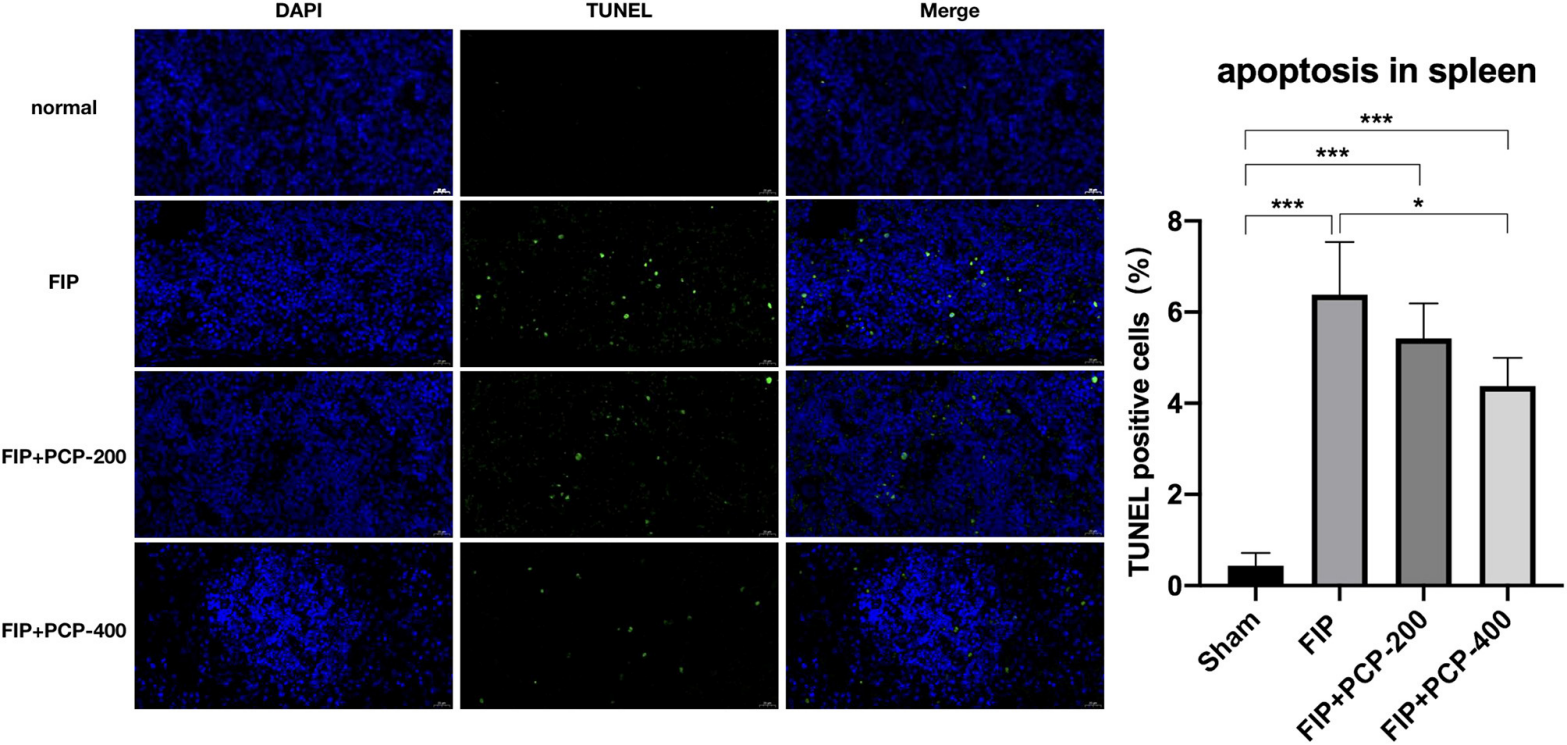
The impact of PCP on vital signs in the fecal peritonitis-induced septic spleen. PCP pretreatment contributed to decreased percentage of TUNEL-positive cells, ^*^P < 0.05; ^***^P < 0.001, FIP + PCP-200 = FIP with PCP 200 mg/kg; FIP + PCP-400 = FIP with PCP 400 mg/kg. All presented data are a composite of three independent experiments. *n* = 5 in each group.

To detect the level of splenocyte cell apoptosis in the FIP mouse in this study, a TUNEL assay of splenic tissue was performed. The fluorescence assay showed that TUNEL-positive cells were sporadically found in the Sham-treated mice, and the numbers significantly increased after the induction of septic peritonitis. TUNEL-positive cells were fertile in the FIP group, while significantly reduced in the PCP-pretreatment group. The apoptotic rate of splenocyte cells was reduced from 7.23 to 3.76% with PCP-400 mg/kg/day pretreatment (Figure 8).

## Discussion

Notably, many Chinese and East-Southeast Asia residents take traditional medicinal materials, such as “Poria Cocos polysaccharide,” daily to strengthen the body's resistance (Wu et al., 2018). We verified the protective effect of PCP on FP-induced sepsis, which has important practical significance for East-Southeast Asia residents. This study found that the mice fed with PCP could better resist the feces and improve mice's clinical score and survival rate. A further study of this effect showed that the inflammation in mice was lower and there were fewer cytokines promoting inflammation when exposed to fecal. Meanwhile, pretreatment with PCPs, the level of oxidative stress was controlled well, and reactive oxygen species (ROS) were reduced. Then, the splenic injury was gentle, the apoptosis of splenocytes was less, the expression of Treg was reduced, and the splenic function to resist damage-associated molecular patterns (DAMPs) injury was maintained. This study provides a new concept for relieving FP-induced sepsis, given the feeble pretreatment regimen for sepsis based on the anti-inflammatory and protective effects of PCP. As a previous report, this study also compared different dose concentrations of PCP to verify the efficacy of different concentrations and suggested that the efficacy of 400 mg/kg/day PCP was better than that of 200 mg/kg/day.

Predominantly, sepsis is considered to be an acute inflammatory state mediated by the activation of the innate immune system caused by infection (Grondman et al., 2020). Molecules derived from pathogens, also known as pathogen-associated molecular patterns (PAMPs), can bind to pattern recognition receptors (PRRs) to activate innate immune cells to protect the host. The activation of PRRs on innate or non-immune cells can trigger intracellular signal cascades, thus activating transcription factors (Wang and Wang, 2013). Intracellular signaling cascades initiate multiple cellular processes, including cytokine production. There are many different PRRs subsets on immune cells (mainly monocytes/macrophages) and non-immune cells that recognize different classes of microorganisms, leading to immune activation (Mahla et al., 2013). Early stage sepsis, the initial hyperinflammatory phase, also known as the “cytokine storm,” is ‘described by the overwhelming release of inflammatory molecules by the innate immune system (Chaturvedi et al., 2021). Hyperinflammation is to induce the initial cause of cell impairment, causing necrotic cells and tissue injury from the original insult and inflammatory reaction (Gustine and Jones, 2021). The TNF-α is an endogenous pyrogen involved in the induction of fever, apoptotic cell death, and inflammation. IL-6 is an important mediator that acts as a pro-inflammatory cytokine (Lonsdale et al., 2020). Meanwhile, the body promotes the secretion level of inflammatory factors (TNF-α, IL-6, and IL-1β) and decreases the secretion level of inflammatory factors, making the body's immune system in a state of immune hyperactivity and cope with the pathogens (London et al., 2010). While this state of affairs continued, the body has been high pro-inflammation factors, and the body's immune cells appear to reduce the number of cells and loss of function to make the body's immune chaos and paralysis, accelerating the death of patients (van der Poll et al., 2021). Therefore, controlling the excessive inflammation in the early stage is significant in sepsis (Bosmann and Ward, 2013). After 14 days of feeding with PCP, mice were intraperitoneally injected with excrement and sacrificed 24 h later. Compared with the mice without feeding PCP, the secretion levels of promoting inflammatory factors were reduced. It indicates that daily administration of PCP can resist the FP and has a good protective effect.

Oxidative stress is a result of an imbalance between the formation of reactive oxidizing species, such as superoxide, hydrogen peroxide, and hydroxyl ions as free radicals, and the removal of antioxidant scavengers, such as catalase, SOD, vitamin C, vitamin E, and reduced GSH (Pizzino et al., 2017; Usmani et al., 2021). In septic conditions, the body needs more energy to sustain life activities than it normally does. Mitochondria generate a large number of oxygen free radicals while providing energy through the electron transport chain (Ramirez-Zuniga et al., 2021). In a physiological state, mitochondria can avoid the damage of ROS by the antioxidant enzymes, such as SOD and GSH. As one of the most important oxygen free radicals in the ROS family, MDA is the main degradation product of lipid peroxidation. By attacking the unsaturated fatty acids in the cell membrane, MDA inhibits the function of membrane protein and leads to the destruction of the integrity of cell membrane and mitochondrial membrane, thus increasing the permeability of cell membrane and leading to cell edema and necrosis. MPO is a member of the heme peroxidase superfamily (Denning et al., 2019; Liu et al., 2020). Its main function is to kill microorganisms in phagocytes and form oxidizing free radicals to participate in many processes of regulating inflammatory responses. When inflammation occurs in the body, the production of MPO increases, resulting in cell damage (Ward and Fattahi, 2019), while higher levels of MPO, on day 1, were able to predict 90-day mortality (Bonaventura et al., 2020). After 14 days of feeding PCP, the MDA and MPO levels were maintained at low levels, and the expressions of GSH and SOD were higher.

People with asplenia or hyposplenism are at an increased risk of fulminant sepsis which carries a high mortality rate (Kanhutu et al., 2017), and it is proved that splenic function is particularly important for patients with sepsis. Splenic cellular histopathology is one of the direct methods to evaluate the splenic injury, and these outcomes were consistent with the results of improved cytoarchitecture in H&E staining (Suttie, 2006; Coccolini et al., 2019). FoxP3^+^ regulatory T cells (Treg), a mature T cell subset with regulatory functions, play an important role in immune homeostasis, allergic reaction, tumor immunity, and transplantation tolerance (Kurup et al., 2017). Treg is a good candidate owing to explaining the impairment of the CD4^+^ T-cell compartment observed during sepsis due to their highly suppressive function (Qureshi et al., 2011). Moreover, the number of Treg cells has upregulated in early-stage patients with sepsis (Leng et al., 2013). Therefore, Treg cells might be contributed to the higher morbidity and mortality in patients with sepsis (Xu et al., 2020), and the role of Treg cells in the immune response to infection has attracted wide attention (Nascimento et al., 2017). While the first line defenders are combating infection, Treg cells respond to infection by suppressing excessive immune responses elicited by other cells of the adaptive immune system, in turn, further shaped by increased numbers of Treg cells (Huang et al., 2010). In current findings from this study, the expression of Treg cells significantly increased after FP-induced sepsis; nevertheless, decreased FoxP3-labeled cells in FP-induced sepsis and PCP pretreated mice were observed.

Until recently, apoptosis is the best-known form of programmed cell death and resulted in the premature death of cells in living tissue by autolysis (Moreno-Gonzalez et al., 2016); some studies have shown that immune cell apoptosis is directly related to the pathogenesis (Huang et al., 2019; Chen and Wei, 2021) and is the major mechanism of lymphocyte death, which is one of the reasons for the gradual decrease in the number of immune cells in sepsis (Aziz et al., 2014). Thereby, a functionally regulated apoptotic program in thymus and splenic cells may provide a promising strategy for combating FP-induced sepsis. Thereby, we reasoned that the anti-apoptotic biological activity of PCP might be one of the underlying molecular mechanisms of protective effects against FP-induced sepsis.

Taken together, our current findings disclose that PCP exerts a promising immunity protective effect against FP-induced sepsis, suggesting PCP's potential perspective of clinical application. The current findings showed that FP-induced and PCP-pretreated sepsis mice resulted in lower TNF-α, IL-6, and IL-1β contents in plasma, downregulating expression of Treg cells in the spleen. Consequently, we concluded that PCP pretreatment inhibited MPO and MDA release, eventually blocking apoptosis. Furthermore, the underlying molecular mechanisms associated with PCP-mediated cytoprotection warrant should be discussed, respectively.

## Limitations

This study has several limitations. First, the concentration of PCP is the key factor to determine the pharmacological effect *in vivo*, and we did not determine under the present condition. Second, we only injected with 1 mg/g of feces according to previous reports and did not conduct relevant studies on dose gradient. The number of feces may have imparity pathophysiological. Our results only suggest that PCP 400 mg/kg/day is effective in preventing sepsis caused by 1 mg/g FP-induced sepsis. Finally, although many people in East Asia have been taking PCP orally for a long time, PCP was fed to mice 14 days in advance. Therefore, these results only suggest that PCP has certain anti-inflammatory and protective effects on FIP-induced mice. The relevant studies on the therapeutic effects of PCP on mice with fecal peritonitis-induced sepsis were absent, and we will continue to do relevant studies. Finally, although some evidence has emerged, we have not been able to connect the evidence into a complete pathway to a specific mechanism and block or overexpression of relevant upstream and downstream pathways, and further studies are needed to explore the specific mechanism of these pharmacological effects.

## Conclusion

The results in this study suggest that PCPs have pharmacological activity against FP-induced sepsis, by the potential molecular mechanism of inhibiting splenic cell apoptosis, inflammatory stress, and oxidative stress.

## Data Availability Statement

The original contributions presented in the study are included in the article/supplementary material, further inquiries can be directed to the corresponding author.

## Ethics Statement

The animal study was reviewed and approved by the Institutional Animal Care and Use Committee at Naval Medical University.

## Author Contributions

YW performed mostly experiments, analyzed the data, and drafted the manuscript. DL and HW analyzed the data and critically revised the manuscript. DL contributed to the data analysis and interpretation. XW conceived and designed the study. All authors substantially contributed to editing, revising, and finalizing the manuscript and read and approved the final manuscript.

## Funding

This work was supported by the Shanghai Natural Science Foundation (19ZR1456600), the Shanghai Pujiang Program (2020PJD059), the Youth Cultivation Project for Special Basic Medical Research of the First Affiliated Hospital of Naval Medical University (2021JCQN02), and the Incubator Program for Startups of the 980th Hospital of the Joint Logistic Support Force (FYJHMS-04).

## Conflict of Interest

The authors declare that the research was conducted in the absence of any commercial or financial relationships that could be construed as a potential conflict of interest.

## Publisher's Note

All claims expressed in this article are solely those of the authors and do not necessarily represent those of their affiliated organizations, or those of the publisher, the editors and the reviewers. Any product that may be evaluated in this article, or claim that may be made by its manufacturer, is not guaranteed or endorsed by the publisher.

## Abbreviations

PCP: Poria Cocos polysaccharide
FIP: fecal-induced peritonitis
GPx: glutathione peroxidase
SOD: superoxide dismutase
MDA: malondialdehyde
MPO: myeloperoxidase
ICU: intensive care unit
TUNEL: dUTP-biotin nick end labeling
ANOVA: analysis of variance
MSS: Murine Sepsis Score
H&E: hematoxylin-eosin staining
DAMPs: damage-associated molecular patterns
ROS: reactive oxygen species
PAMPs: pathogen-associated molecular patterns
PRRs: pattern recognition receptors
SABC: strept avidin biotin complex.
